# The Distribution of Charged Amino Acid Residues and the Ca^2+^ Permeability of Nicotinic Acetylcholine Receptors: A Predictive Model

**DOI:** 10.3389/fnmol.2017.00155

**Published:** 2017-05-29

**Authors:** Sergio Fucile

**Affiliations:** ^1^Department of Physiology and Pharmacology “V. Erspamer”, Sapienza Università di RomaRome, Italy; ^2^Molecular Pathology, Istituto Neurologico Mediterraneo (IRCCS), Parco TecnologicoPozzilli, Italy

**Keywords:** fractional Ca^2+^ current, ion selectivity, nicotinic subunits, charged amino acids, nicotine dependence

## Abstract

Nicotinic acetylcholine receptors (nAChRs) are cation-selective ligand-gated ion channels exhibiting variable Ca^2+^ permeability depending on their subunit composition. The Ca^2+^ permeability is a crucial functional parameter to understand the physiological role of nAChRs, in particular considering their ability to modulate Ca^2+^-dependent processes such as neurotransmitter release. The rings of extracellular and intracellular charged amino acid residues adjacent to the pore-lining TM2 transmembrane segment have been shown to play a key role in the cation selectivity of these receptor channels, but to date a quantitative relationship between these structural determinants and the Ca^2+^ permeability of nAChRs is lacking. In the last years the Ca^2+^ permeability of several nAChR subtypes has been experimentally evaluated, in terms of fractional Ca^2+^ current (*Pf*, i.e., the percentage of the total current carried by Ca^2+^ ions). In the present study, the available *Pf*-values of nAChRs are used to build a simplified modular model describing the contribution of the charged residues in defined regions flanking TM2 to the selectivity filter controlling Ca^2+^ influx. This model allows to predict the currently unknown *Pf*-values of existing nAChRs, as well as the hypothetical Ca^2+^ permeability of subunit combinations not able to assemble into functional receptors. In particular, basing on the amino acid sequences, a *Pf* > 50% would be associated with homomeric nAChRs composed by different α subunits, excluding α7, α9, and α10. Furthermore, according to the model, human α7β2 receptors should have *Pf*-values ranging from 3.6% (4:1 ratio) to 0.1% (1:4 ratio), much lower than the 11.4% of homomeric α7 nAChR. These results help to understand the evolution and the function of the large diversity of the nicotinic receptor family.

## Introduction

Ca^2+^ ions are able to permeate through the cation-selective pentameric nicotinic acetylcholine receptors (nAChRs; Katz and Miledi, 1969; Bregestovski et al., 1979; Eusebi et al., 1980; for review see Fucile, 2004). This Ca^2+^ entry pathway plays relevant physiopathological roles, for instance positively modulating neurotransmitter release in neuronal presynaptic terminals (Wonnacott, 1997) or damaging the muscle endplates in patients with slow-channel congenital myasthenic syndromes (Engel et al., 2003). For these reasons the quantification of the amount of Ca^2+^ flowing through a particular nAChR subtype is a relevant goal to understand its functional role and the physiological consequences of its activation. The very first methodological approach to quantitatively determine the ion channel Ca^2+^ permeability was based on the measurement of the reversal potential shift upon changes in [Ca^2+^]_o_, and then calculating the *P*_*Ca*_/*P*_*Na*_ ratio using the Goldman-Hodgkin-Katz constant field assumptions (Lewis, 1979). A second experimental approach was later introduced by Erwin Neher and his group in the early 90's, basing on the possibility to simultaneously record transmembrane currents with the Patch-Clamp techniques and the [Ca^2+^]_i_ changes with fluorescence microscopy (Zhou and Neher, 1993; Neher, 1995), and leading to the direct measurement of the fractional Ca^2+^ current, usually indicated as *Pf* and representing the percentage of the total current carried by Ca^2+^ ions. A careful comparison of the two methods (Burnashev et al., 1995) indicated that the second one was more advisable, being independent from the constant field assumptions, not always respected in real ion channels. In the last two decades the Ca^2+^ permeability of several nAChRs has been characterized in terms of *Pf*, using the Neher's methodological approach (see references in **Table 2** and in Fucile, 2004). These studies highlighted a large variability of Ca^2+^ permeability, with *Pf*-values ranging from 1.5% (human α4β4; Lax et al., 2002) to 22% (rat α9α10; Fucile et al., 2006b), and raise questions about the physiological meaning and the structural determinants of this large spectrum. In particular, seminal studies have clearly indicated a fundamental role of charged amino acid rings flanking the transmembrane region TM2, for single-channel conductance (Imoto et al., 1988), cation selectivity (Corringer et al., 1999), and Ca^2+^ permeation (Bertrand et al., 1993). It is widely known and accepted that these highly conserved charged residues, when mutated, may profoundly alter the ion selectivity filter of nAChRs, leading to significant changes of ion permeability (Bertrand et al., 1993; Corringer et al., 1999). Despite this long established knowledge, the possibility to quantitatively relate the Ca^2+^ permeability to the presence of charged residues in key positions is lacking. Though in theory it is possible to build a molecular model to simulate the channel energy profile and numerically calculate the Ca^2+^/Na^+^ flow ratio for each distinct nAChR subtype (Song and Corry, 2009), this approach would be extremely onerous. In this study the sum of the electrical charges present in different regions of the channels have been considered as modular components of the selectivity filter and quantitatively analyzed, with the aim to understand the contribution of each region to the Ca^2+^ influx through nAChRs, and to build a quantitative model able to predict *Pf*-values from the amino acid sequences of nAChR subunits.

## Methods

The amino acid sequences of all nicotinic subunits considered in this study have been obtained by the Uniprot website, and all the Uniprot accession numbers are given in Table 1. For each pentameric subunit assembly considered, the sum of electrical charges arising from negative (aspartate, glutamate) and positive (arginine, histidine, lysine) residues has been calculated in different portions of the channel. In particular each histidine was considered contributing +0.1 in the extracellular solution (pH 7.4) or +0.4 in the intracellular one (pH 7.0). The channel sections considered in the study were: (1) the extracellular region, composed by both N- and C-terminal side of the five subunits; (2) the intracellular region between TM3 and TM4 transmembrane regions; (3) the {−5′ −4′} positions in the intracellular side of TM2 (where 0′ is the conserved lysine residue immediately preceding TM2, according to Miller, 1989); (4) the {−1′} position; (5) the {19′} position in the extracellular side of TM2; (6) the {20′} position; (7) the {21′–24′} positions; (8) the {25′–29′} positions. The fractional Ca^2+^ current (*Pf*, i.e., the percentage of the total current carried by Ca^2+^ ions) values were obtained from previous measurements made in our laboratory, in the same (or very similar) experimental conditions (for details see references in Table 2). When considering experiments in which the transfection of two subunits could result in the coexpression of two alternative stoichiometry, the mean charge distribution has been used. All the information used to build the model are reported in Table 1 (subunits) and in Table 2 (assembled receptors).

**Table 1:** **Sequences of amino acids near and in TM2 transmembrane regions of 28 nAChR subunits**.

**sp**.	**sub**.	**Uniprot**			**TM2**				
			**{−5′− 4′}**	**{−3′ 0′}**	**{1′ 18′}**	**{19′}**	**{20′}**	**{21′ 24′}**	**{25′ 29′}**
H	α1	P02708	DS	_GEK	MTLSISVLLSLTVFLLVI	V	E	LIPS	TSSAV
H	β1	P11230	DA	_GEK	MGLSIFALLTLTVFLLLL	A	D	KVPE	TSLSV
H	γ	P07510	KA	GGQK	CTVAINVLLAQTVFLFLV	A	K	KVPE	TSQAV
H	δ	Q07001	DS	_GEK	TSVAISVLLAQSVFLLLI	S	K	RLPA	TSMAI
H	ε	Q04844	QA	GGQK	CTVSINVLLAQTVFLFLI	A	Q	KIPE	TSLSV
M	α1	P04756	DS	_GEK	MTLSISVLLSLTVFLLVI	V	E	LIPS	TSSAV
M	β1	P09690	DA	_GEK	MGLSIFALLTLTVFLLLL	A	D	KVPE	TSLAV
M	γ	P04760	KA	GGQK	CTVATNVLLAQTVFLFLV	A	K	KVPE	TSQAV
M	δ	P02716	DC	_GEK	TSVAISVLLAQSVFLLLI	S	K	RLPA	TSMAI
M	ε	P20782	QA	GGQK	CTVSINVLLAQTVFLFLI	A	Q	KIPE	TSLSV
H	α2	Q15822	DC	_GEK	ITLCISVLLSLTVFLLLI	T	E	IIPS	TSLVI
H	α3	P32297	DC	_GEK	VTLCISVLLSLTVFLLVI	T	E	TIPS	TSLVI
H	α4	P43681	EC	_GEK	ITLCISVLLSLTVFLLLI	T	E	IIPS	TSLVI
H	α5	P30532	NE	_GEK	ICLCTSVLVSLTVFLLVI	E	E	IIPS	SSKVI
H	α6	Q15825	DC	_GEK	VTLCISVLLSLTVFLLVI	T	E	TIPS	TSLVV
H	α7	P36544	DS	_GEK	ISLGITVLLSLTVFMLLV	A	E	IMPA	TSDSV
H	β2	P17787	DC	_GEK	MTLCISVLLALTVFLLLI	S	K	IVPP	TSLDV
H	β4	P30926	DC	_GEK	MTLCISVLLALTFFLLLI	S	K	IVPP	TSLDV
M	α4	O70174	EC	_GEK	VTLCISVLLSLTVFLLLI	T	E	IIPS	TSLVI
M	α5	Q2MKA5	NE	_GEK	ISLCTSVLVSLTVFLLVI	E	E	IIPS	SSKVI
M	β2	Q9ERK7	DC	_GEK	MTLCISVLLALTVFLLLI	S	K	IVPP	TSLDV
R	α7	Q05941	DS	_GEK	ISLGITVLLSLTVFMLLV	A	E	IMPA	TSDSV
R	α9	P43144	AS	_GEK	VSLGVTILLAMTVFQLMV	A	E	IMPA	SENVP
R	α10	Q9JLB5	DS	_GEK	VSLGVTVLLALTVFQLIL	A	E	SMPP	AESVP
C	α3	P09481	DC	_GEK	VTLCISVLLSLTVFLLVI	T	E	TIPS	TSLVI
C	α4	P09482	EC	_GEK	ITLCISVLLSLTVFLLLI	T	E	IIPS	TSLVI
C	β2	P09484	DC	_GEK	MTLCISVLLALTVFLLLI	S	K	IVPP	TSLDV
C	β4	P26153	DC	_GEK	MTLCISVLLALTVFLLLI	S	K	IVPP	TSLDV

*Negative amino acid residues are reported in red, positive ones in green. H, human; M, mouse; R, rat; C, chicken. In brackets the positions of amino acid residues according to Miller (1989)*.

**Table 2:** **Charge distribution and measured ***Pf***-values of the indicated 16 nAChRs**.

**sp**.	**Type**	**Ext. domain**			**TM2**				**Int**.	***Pf (%)***	**References**
			**{−5′−4′} cytopl. ring**	**{−1′} inter. ring**	**{19′}**	**{20′} extrac. ring**	**{21′ 24′}**	**{25′ 29′}**	**TM3 TM4**		
H	α4β4	−12.75	−5	−5	0	0	0	−2.5	36.5	1.5 ± 0.2	Lax et al., 2002
M	αβγδ	−63	−3	−4	0	−1	1	0	22.6	2.1 ± 0.3	Ragozzino et al., 1998
C	α4β4	−17.25	−5	−5	0	0	0	−2.5	35	2.1 ± 0.2	Lax et al., 2002
H	α4β2	−34.25	−5	−5	0	0	0	−2.5	37	2.6 ± 0.3	Lax et al., 2002
H	α3β4	−18.75	−5	−5	0	0	0	−2.5	26.5	2.7 ± 0.2	Lax et al., 2002
H	αβγδ	−53	−3	−4	0	−1	1	0	23.4	2.9 ± 0.2	Fucile et al., 2006a
C	α4β2	−25	−5	−5	0	0	0	−2.5	46.5	2.9 ± 0.5	Ragozzino et al., 1998
H	(β2α4)_2_α4	−31.7	−5	−5	0	−1	0	−2	39	4.0 ± 0.4	Sciaccaluga et al., 2015
M	αβεδ	−69	−4	−4	0	−2	1	0	15.2	4.2 ± 1.0	Ragozzino et al., 1998
C	α3β4	−23	−5	−5	0	0	0	−2.5	4.5	4.4 ± 0.5	Lax et al., 2002
H	αβεδ	−55.9	−4	−4	0	−2	1	0	17.8	7.2 ± 1.1	Fucile et al., 2006a
H	(β2α4)_2_α5	−32.1	−5	−5	−1	−1	0	−1	35	8.2 ± 0.7	Sciaccaluga et al., 2015
M	(β2α4)_2_α5	−32	−5	−5	−1	−1	0	−1	43.6	8.8 ± 1.1	Sciaccaluga et al., 2015
R	α7	−23.5	−5	−5	0	−5	0	−5	18	8.8 ± 1.5	Fucile et al., 2003
H	α7	−23	−5	−5	0	−5	0	−5	16	11.4 ± 1.3	Fucile et al., 2003
R	α9α10	−40.75	−2.5	−5	0	−5	0	−5	55.5	22 ± 4	Fucile et al., 2006b

*H, human; M, mouse; R, rat; C, chicken. In brackets the positions of amino acid residues according to Miller (1989). Pf, fractional Ca^2+^ current. Charge values are expressed in natural unit of electric charge (nuec)*.

### Fitting procedure

To evaluate the contribution of charged amino acids placed in different region of nicotinic subunits to the Ca^2+^ permeability of distinct nAChRs, a very simple methodological approach has been followed. In the presence of a single energy barrier ΔG, if all other parameters are fixed (membrane potentials, temperature), and if the flowing ions are present only at the outside of the membrane at fixed concentration, the flowing current will be proportional to e^−ΔG/RT^ (Hille, 2001).

In these conditions,

(1) $$\begin{array}{l} I_{Ca}\;=\;C_{Ca}{\;e}^{-\frac{△G_{Ca}}{RT}} \end{array}$$

where the term *C*_*Ca*_ is constant when ion concentrations, potential and temperature are fixed, and Δ*G*_*Ca*_ is the energy barrier value for Ca^2+^ ions.

The same relation is valid for Na^+^ fluxes:

(2) $$\begin{array}{l} I_{Na}\;=\;C_{Na}{\;e}^{-\frac{△G_{Na}}{RT}} \end{array}$$

Given that, when Na^+^ and Ca^2+^ are the only permeant ions, by definition

(3) $$\begin{array}{l} P_{f}\;=\;\frac{I_{Ca}}{I_{Ca}\;+\;I_{Na}} \end{array}$$

then

(4) $$\begin{array}{l} P_{f}\;=\;\frac{C_{Ca}\;e^{-\frac{△G_{Ca}}{RT}}}{C_{Ca}\;e^{-\frac{△G_{Ca}}{RT}}+\;C_{Na}\;e^{-\frac{△G_{Na}}{RT}}}\;=\;\frac{1}{1\;+\;\frac{C_{Na}}{C_{Ca}}\;e^{\frac{△G_{Ca}-△G_{Na}}{RT}}} \end{array}$$

With a strong oversimplification, the term Δ*G*_*Ca*_ – Δ*G*_*Na*_ has been substituted by a weighted sum of the electric charges (Δ*q*_*i*_) associated to the negative (glutamate, aspartate) and positive (lysine, arginine, histidine) amino acids present in 1, 2, 3, or 5 (n) different regions of the nicotinic subunits. Furthermore, the constant ratio *C*_*Na*_/*C*_*Ca*_ has been substituted by a single constant *const*. Thus, *P*_*f*_ data have been fitted with the following equation:

(5) $$\begin{array}{l} P_{f}\;=\;\frac{1}{1\;+\;const\;e^{\sum_{i\;=\;1}^{n}k_{i}△q_{i}}} \end{array}$$

For each fit the *R*^2^-value has been reported, and the predicted *Pf*-values have been compared with the real ones. The *const* and *k*_*i*_-values obtained by fitting the charge distribution in the five out the eight regions were then used to predict the *Pf*-values of several pentameric subunit combination giving rise to unreal or existing nicotinic receptors. The natural unit of electrical charge (nuec) has been used as charge unit throughout the paper.

## Results

**Figure 1:**
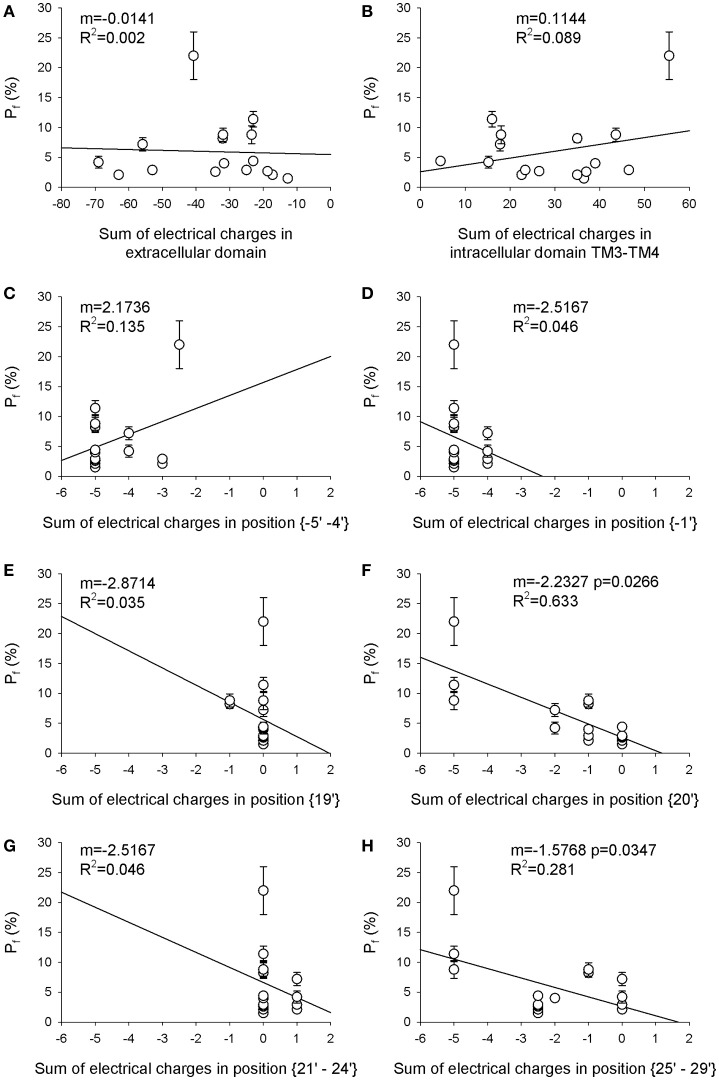
**Electrical charge in the TM2 extracellular ring (position 20′) is the main determinant of Ca^**2+**^ permeability**. The sum of the electrical charges associated with charged amino acids in eight different regions of each nAChR reported in Table 1 has been calculated and plotted against the *Pf*-value of the same nAChR. **(A)** extracellular domain; **(B)** intracellular domain between TM3 and TM4; **(C)** position {−5′ −4′} (where 0′ is the conserved lysine residue immediately preceding TM2, according to Miller, 1989); **(D)** position {−1′}; **(E)** position {19′}; **(F)** position {20′}; **(G)** position {21′–24′}, **(H)** position {25′–29′}. In each panel the angular coefficient m of the linear regression is reported, expressed in nuec^−1^, as well as the *R*^2^-value, to evaluate the fit. Please note the significant linear correlations reported in **(F)** for position {20′}, and in **(H)** for position {25′–29′}, with the indicated *p*-values.

The first aim of this study was to verify if the Ca^2+^ permeability of 16 different nAChRs (measured in terms of fractional Ca^2+^ current, *Pf*) correlated with the local distribution of the electrical charges associated to the amino acid sequences of their subunits (Table 1). The first region analyzed was the extracellular domain composed by both the large N-terminal and the short C-terminal segments. A net negative charge characterizes the extracellular domains of all nAChRs, with a clear segregation of muscle receptors, exhibiting a stronger negativity comprised between −50 and −70, while in all other nAChRs the charge sum was comprised between −10 and −40 (Table 2 and Figure 1A). By contrast, in the intracellular regions composed by the five segments between transmembrane helices TM3 and TM4, there was a large prevalence of positive charges (Table 2 and Figure 1B), likely contributing to interaction sites with intracellular negative molecules. Linear regressions were used to estimate the correlation between observed *Pf* and charge values, and the resulting coefficients and statistics indicated for both intracellular and extracellular regions the absence of any correlation. These portions of the receptor-channel were no longer considered for further analysis in the present study. Then the charges near the TM2 helices were analyzed to look for significant correlations with Ca^2+^ permeability. Six distinct locations were chosen, forming two intracellular rings, i.e., position {−5′ −4′}, and the position {−1′}, or four extracellular rings: position {19′}; position {20′}; position {21′ 24′}; position {25′ 29′}. It is worth to note some characteristic features of the charge distribution described in Tables 1, 2 and in Figure 1: (a) in position {20′} α subunits always exhibit a negative charge, while positive charges are present in most non-α subunits; (b) position {19′} exhibits a negative charge only when an α5 subunit is present; (c) the charge distribution of position {−1′} and position {21′ 24′} are overlapping, yielding the same linear regression results, due to the fact that the muscle receptors in both position exhibit a one-unit more positive charge than all other receptors. This last observation makes the two positions redundant, and in the following analysis only position {−1′} will be considered. The data and the regressions reported in Figures 1C–H clearly indicated significant negative correlations when considering charges in position {20′} (Figure 1F) or in position {25′29′} (Figure 1H), with *p* < 0.05 for angular coefficients. In other positions the linear regressions still yielded negative angular coefficients, supporting the notion that in general the increase of the overall negative charge corresponds to an enhancement of Ca^2+^ fluxes, with the only exception of position {−5′ −4′}.

**Figure 2:**
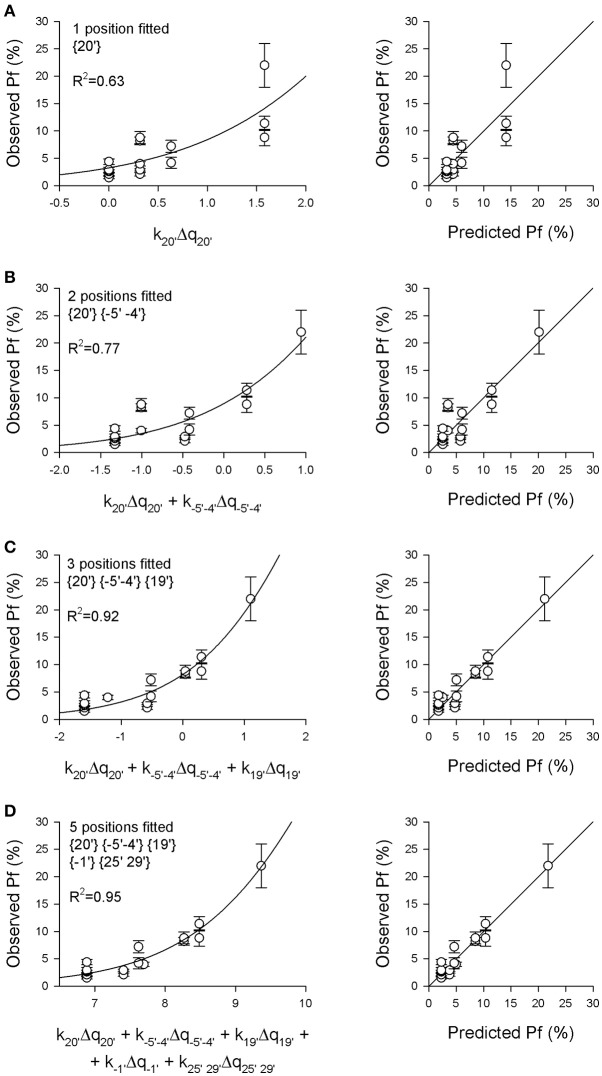
**The ***Pf***-values of 16 different nAChRs are predicted by a model based on the charge distributions in five distinct pore regions**. The experimentally measured *Pf*-values reported in Table 2 were plotted against the weighted sum of the electrical charges Δq_*i*_ present in different pore regions, as indicated, with the multiplicative k_*i*_-values allowed to vary in order to best fit the data, according to Equation $P_{f}=\frac{1}{1\;+\;const\;e_{i=1}^{\sum n}k_{i}△q_{i}}$ (see Section Methods for details). **(A)** Best fit of *Pf*-values (left) and comparison between observed and predicted *Pf*-values (right), using as independent variable only the electrical charge present in the extracellular position {20′}, representing the main determinant of Ca^2+^ permeability (const = 29.5, *p* = 0.0029; k_20′_ = −0.316, *p* = 0.0003). **(B)** The charge distributions in position {20′} and in the intracellular position {−5′ −4′} were used as independent variables (const = 10.2, *p* = 0.0501; k_20′_ = −0.321, *p* = 0.0001; k_−5′−4′_ = 0.266, *p* = 0.013). **(C)** The charge distributions in positions {20′}, {−5′ −4′} and {19′} were used as variables (const = 11.2, *p* = 0.0084; k_20′_ = −0.3806, *p* < 0.0001; k_−5′−4′_ = 0.3197, *p* = 0.0002; k_19′_ = −1.260, *p* = 0.0002). Note the substantial improvement of the fit adding the charges in position {19′} to the analysis, which now takes into account the negative glutamate residue present in α5 subunits in this position. **(D)** The electrical charges of the five indicated positions were used as variables (const = 41917, *p* = 0.82; k_20′_ = −0.5654, *p* = 0.0134; k_−5′−4′_ = 0.3521, *p* < 0.0001; k_19′_ = −0.0798, *p* = 0.90; k_−1′_ = −1,975, *p* = 0.0816; k_25′ 29′_ = 0.491, *p* = 0.1598). Note the high *R*^2^-value and the excellent agreement between observed and predicted *Pf*-values.

The second aim of this study was to quantitatively estimate the contribution of the charges distributed in different receptor regions to the nAChR Ca^2+^ permeability, to have a better understanding of the structural determinants of this relevant physiological parameter, and to try to build a simple model allowing to predict the effect of different subunit compositions or mutations on the *Pf*-value. Thus, a progressive multidimensional fitting procedure was adopted (for details see Section Methods), in which the sigmoidal function described by Equation (5) was used to best fit the 16 known *Pf*-values, starting using as independent variable the sum of electrical charges in position {20′}, where it was observed the most significant correlation. Successive fit procedures used the electrical charges present in two or more positions as independent variables, progressively choosing the positions giving the higher *R*^2^-values. In Figure 2 the sigmoidal curves best fitting the *Pf*-values are plotted using as abscissa the weighted sum of the charges in different receptor regions, where the *k*_*i*_ constant were derived from the fitting procedure. For each fit the comparison between observed and predicted *Pf*-values is reported. As expected from the linear regression analysis, the best monodimensional fit was obtained using the position {20′} data as independent variable (Figure 2A). However, this charge distribution alone is not able to explain large differences in Ca^2+^ permeability. When adding a second independent variable, the best result was obtained with position {−5′ −4′}, differently from what could be expected from linear analysis. This effect may be due to the similar pattern of charge-receptor association exhibited by both position {20′} and position {25′ 29′} (Figures 1F,H), not really helping to discriminate between different receptors. By contrast, the introduction of the data of position {−5′ −4′} clearly helped to discriminate between highly Ca^2+^ permeable nAChRs (e.g., α7 vs. α9α10 nAChRs; Figure 2B). The best fit result in terms of statistical significance was obtained with three independent variables, i.e., the charge distributions of position {20′}, position {−5′ −4′} and position {19′} (Figure 2C). Including all these data, the *Pf* of α5-containing nAChRs were much better accounted for, the *R*^2^-value was strongly enhanced and all the *k*_*i*_ constants were statistically significant (*p* < 0.05, see legend of Figure 2 for details). However, several subunit combinations present distinct charge patterns in the other locations, thus a final fit based on all five independent regions was made, providing the best correlation between observed and predicted *Pf*-values (Figure 2D; *R*^2^ = 0.95).

The resulting *k*_*i*_ constants values, reported in Table 3, were then used to weight the relative contribution of each position and to build a simple modular model linking the electrical charges present in the examined amino acid rings to a *Pf*-value. In this way it was possible to predict the hypothetical *Pf*-values of several combinations of human subunits which are not able to assemble into functional nAChRs in living cells (in red in Table 3), but also of existing receptors whose Ca^2+^ permeability has not yet experimentally measured (in green in Table 3). The proposed model indicates very high *Pf*-values (>50%) for homomeric nAChRs constituted by α subunits (from α1 to α6), all lacking the five negative charges in position {25′ 29′} in the α7 subunit, which determine for this last receptor the reduction to 10.4%. Interestingly, if the charges in position {−1′} are removed, as for α7_E237A_, the *Pf*-value is 0.0, exactly as expected from the direct measure of Ca^2+^ permeability reported for this mutated nAChR (Bertrand et al., 1993). The model has been applied to the α7β2 nAChRs (Wu et al., 2016), which may be expressed with different subunit ratios. The higher the number of β2 subunits, the lower the Ca^2+^ permeability, due to the increasing positivity in position {20′}. Other heteromeric combination are reported, with higher values for combinations with α5 subunit. Thus, the proposed model suggests a dramatically high Ca^2+^ permeability for non-existing homomeric nAChRs likely eliminated during evolution, describes the effect on Ca^2+^ permeability of known mutations, and allows to reasonably predict the *Pf*-values of physiologically relevant heteromeric nAChRs.

**Table 3:** **Charge distribution and predicted ***Pf***-values of human nAChRs with different subunit composition**.

		**TM2**						
	**Position**	**{−5′−4′}**	**{−1′}**	**{19′}**	**{20′}**	**{21′ 24′}**	**{25′ 29′}**	
		**k_−5′ −4′_ = 0.3521**	**k_−1′_ = −1.9747**	**k_19′_ = −0.0798**	**k_20′_ = −0.5654**		**k_25′ 29′_ = 0.4910**	
		**cytoplasmic ring**	**intermediate ring**		**extracellular ring**			
**Homom**.								**Predicted** ***Pf*****-values**
α1		−5	−5	0	−5	0	0	57.4
α2		−5	−5	0	−5	0	0	57.4
α3		−5	−5	0	−5	0	0	57.4
α4		−5	−5	0	−5	0	0	57.4
α5		−5	−5	−5	−5	0	0	66.7
α6		−5	−5	0	−5	0	0	57.4
α7		−5	−5	0	−5	0	−5	10.4
α7_E237A_		−5	0	0	−5	0	−5	0.0
**Heterom**.	**Ratio**							
α7β2	4:1	−5	−5	0	−3	0	−5	3.6
α7β2	3:2	−5	−5	0	−1	0	−5	1.2
α7β2	2:3	−5	−5	0	1	0	−5	0.4
α7β2	1:4	−5	−5	0	3	0	−5	0.1
α2β2	1:1	−5	−5	0	0	0	−2.5	2.3
α3β4α5	2:2:1	−5	−5	−1	−1	0	−1	8.5
α4β2α6	2:2:1	−5	−5	0	−1	0	−2	5.0
α4β2α7	1:2:2	−5	−5	0	−1	0	−4	1.9
α4β2α7	2:2:1	−5	−5	0	−1	0	−3	3.1
α4α5α6β2	1:1:1:2	−5	−5	−1	−1	0	−1	8.5
α6β4	1:1	−5	−5	0	0	0	−2.5	2.3

*Non-existing homomeric nAChRs are indicated in red. Potential heteromeric nAChRs in green. Values for homomeric α7 and α7_E237A_ nAChRs are reported for comparison, in black. In brackets the positions of amino acid residues according to Miller (1989). Pf, fractional Ca^2+^ current. Charge values are expressed in natural unit of electric charge (nuec). K_i_-values (expressed in nuec^−1^) indicate the multiplicative constants obtained by the fitting procedure described in the text, and used to predict the Pf-values*.

## Discussion

The main results of this study are represented by an improved picture of the functional relations between the distribution of electrical charges and Ca^2+^ fluxes in nAChRs and the availability of a new simple model to predict *Pf*-values associated to any (possible or impossible) pentameric combination of nicotinic subunits. Relating known *Pf*-values to the charge spatial design of the corresponding nAChRs provided several observations.

First, the Ca^2+^ permeability of these receptors does not depend on the total amount of charge present in the large extracellular or intracellular domains. All extracellular domains have an overall negative charge, with muscle nAChRs exhibiting the most negative values in this region. The extracellular overall negative charge may be functionally relevant for agonist binding, assembly, posttranslational modification, and interactions with extracellular molecular apparatus, in particular for muscle nAChRs which cooperate with a large number of molecular components at the endplate (Witzemann et al., 2013), but there is no evidence of a role in shaping Ca^2+^ permeability. This finding becomes relevant when considering that Ca^2+^ permeability of nAChRs can be modulated by extracellularly applied drugs, such as verapamil or salbutamol (Piccari et al., 2011), which in theory could act in the large extracellular nAChR vestibule. Analogously, though all intracellular domains formed by the five TM3-TM4 segments exhibit overall positive charges, hosting interactions sites for cytoplasmic factors (Stokes et al., 2015), there is no correlation between *Pf*-values and the amount of electrical charges present at this location.

By contrast, significant relationships between Ca^2+^ permeability and charged residues are evident when considering the regions closer to TM2: in particular the best linear correlation is observed in the extracellular ring, in position {20′}, where increasing negativity is significantly linked to higher *Pf*-values. Interestingly, although a similar relation is evident also in residues slightly farther in respect to TM2, in position {25′ 29′}, in the model arising from the fitting procedure this two regions appear to differently contribute to the Ca^2+^ permeability: negative residues in position {20′}enhance it, while those in position {25′ 29′} reduce it. The same kind of inverse relation is present also in the cytoplasmic ring (position {−5′ −4′}). These results confirm for nAChRs the observation that negatively charged sites may either facilitate Ca^2+^ flow, reducing the electrostatic energy profile for divalent cations, or counteract it, in particular if the negative residues represent a high affinity Ca^2+^ binding site in which Ca^2+^ itself may produce a long lasting electrical repulsion for other incoming divalent cations (Yang et al., 1993).

Other charge distributions are extremely conserved: in particular, both at the intermediate ring and at position {21′ 24′}) only muscle nAChRs exhibit a slight less negative charge than all other receptors. In the intermediate ring a reduction of the negative charge is associated to reduced *Pf*-values, as strongly confirmed by α7_E237A_ homomeric nAChR: this mutation has been shown to abolish Ca^2+^ permeability (Bertrand et al., 1993) and consistently the present model reports only for this mutated nAChRs a zero *Pf*-value.

The α5 subunit presents a unique feature: it is the only nicotinic subunit to host a negative residue in position {19′} and a positive one in position {25′ 29′}, both conferring high Ca^2+^ permeability to α5^*^ nAChRs. A hypothetical α5 homomeric nAChR would exhibit, according to this model, the highest calculated *Pf*-value, and the replacement by an α5 subunit of any other α subunit in a heteromeric nAChR always enhance Ca^2+^ permeability. This finding is of relevant physiological interest, given the role of α5^*^ nAChRs in modulating the self-administration of nicotine and the overall use of tobacco (Fowler et al., 2011). The polymorphic variant α5_D398N_, associated with a higher incidence of smoking and lung cancer (Bierut et al., 2008), does not affect the Ca^2+^ permeability of these receptors, but appears to be differently modulated by Ca^2+^ in the cytoplasmic domain (Sciaccaluga et al., 2015). Given the high Ca^2+^ permeability, an attractive hypothesis is that Ca^2+^ may modulate the same α5^*^ nAChRs which it flows through, with the α5_D398N_ subunit dysfunctional in this process, leading to less nicotine aversion (Frahm et al., 2011) and increased nicotine self-administration.

**Figure 3:**
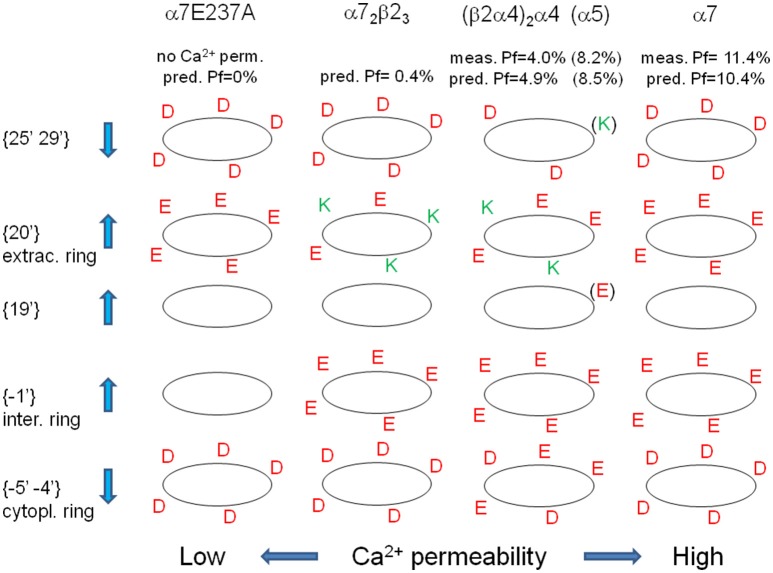
**Spatial distribution of charged amino acid residues in the proximity of the channel pore, for different nAChRs**. For the indicated nAChRs the distribution of positive (green) and negative (red) amino acid residues is reported for each distinct TM2-flanking regions. Up- and down-wards arrows indicate rings in which higher negativity is associated with higher or lower Ca^2+^ permeability, respectively. In brackets the residues present if an α5 subunit replace an α4 subunit, yielding a strong increase in Ca^2+^ permeability. For each nAChR measured and predicted *Pf*-values are reported (in brackets for α5-contaning nAChRs).

All these observations confirm and summarize several previous reports concerning the role of charged amino acids in some of the regions considered in this study (Bertrand et al., 1993; Corringer et al., 1999; Tapia et al., 2007; Sciaccaluga et al., 2015), and allow a comprehensive description of the different selectivity filters exhibited by distinct nAChRs. In summary, it is possible to group nAChRs in three functional categories: Na^+^-selective; Ca^2+^-selective; non-selective between Na^+^ and Ca^2+^. The last ones, in fixed conditions, allow the flow of Ca^2+^ and Na^+^ ions with the same probability, and should exhibit, in 2 mM [Ca^2+^]_i_ and 140 mM [Na^+^]_i_, a *Pf*-value of 2.8%, due to the relative abundance of the two ions. Indeed, many heteromeric nAChRs exhibit *Pf* around this value (see Table 2). Ca^2+^-selective nAChRs favor Ca^2+^ over Na^+^ due to a complex organization of the charges placed in strategic positions: (i) an increased negativity in the immediate intra- and extra-cellular proximity of the channel pore (positions {−1′}, {19′}, and {20′}), with a mechanism similar to the selectivity filter of voltage-gated Ca^2+^ channels (Yang et al., 1993); (ii) a decreased negativity in farther intra- and extra-cellular positions ({−5′ −4′} and {25′ 29′}). The vice-versa is true for Na^+^-selective nAChRs, as for instance α7E237A or α4β4. In Figure 3 the spatial distributions of charged amino acid residues has been shown for several paradigmatic nAChRs, with upward arrows indicating positions in which higher negativity implies higher Ca^2+^ permeability.

The analysis of the predicted *Pf*-values reported in Table 3 leads to interesting observations. First, the surprisingly high Ca^2+^ permeability of all nAChRs ideally formed by non-existing homomeric combinations of different α subunits. All these values are much higher than the *Pf* observed and predicted for homomeric α7 nAChR, suggesting that the exclusion of these receptors could be due to evolutionary processes opposing excessive Ca^2+^ entry and excitotoxicity. In contrast with the usual association “α7-high Ca^2+^ permeability,” this subunit appears to be the α subunit less able to create the conditions for large Ca^2+^ fluxes through nAChRs, and exactly for this reason it could have been allowed by evolution to form homomeric receptors. This hypothesis is supported also by model predictions in which α7 substitutes for a different α subunit in heteromeric nAChRs, as for α4(β2)_2_(α7)_2_ vs. (α4β2)_2_α4 (*Pf*-values of 1.9 vs. 4.0%, respectively). In this view, the insertion in heteromeric neuronal nAChRs of β2 or β4 subunits together with α2, α3, α4, or α6 appears a protective process, adding positive lysine residues instead of negative ones in the extracellular ring (position {20′}). The same role of “Ca^2+^ limiter” is played by β2 subunit in heteromeric nAChRs containing α7 subunits: decreasing the ratio α7:β2 considerably lowers the *Pf*-values of the corresponding nAChRs, suggesting distinct physiological roles for different subunit combinations forming these recently described heteromeric receptors (Wu et al., 2016).

The predictive model presented in this study is based on a strongly oversimplified interpretation of the distribution of charged amino acid residues in selected regions of the receptor-channels, and presents clear limitations. First, it is well-known that the nAChR Ca^2+^ permeability depend also on uncharged residues (Di Castro et al., 2007), and the geometry of the pore, highly relevant for ion-ion and ion-amino acid interactions, has been weakly taken into account, with a literature-based but still arbitrary regional partition. A deeper analysis should use molecular dynamics techniques to numerically build, for each individual nAChR, a proper model taking into account all the interactions able to modulate Ca^2+^ fluxes, in an extremely complex picture.

Furthermore, in the present study the overall extracellular distribution of charged amino acids did not appear to affect Ca^2+^ fluxes through nAChRs, but a single acid residue in the extracellular domain has been shown to strongly decrease the Ca^2+^ permeability of α7 nAChR (rat aspartate 44, corresponding to human aspartate 42; Colón-Sáez and Yakel, 2014), clearly indicating the a more complex analysis would be necessary.

Despite all these known limitations, the present simple approach led to a surprisingly good description of the relation between charge distribution and *Pf*-values, strongly indicating that the amount of negative charges in strategic intracellular and extracellular TM2-flanking regions represents the main determinant of nAChR Ca^2+^ permeability.

This study might be useful as a starting point to build future more sophisticated models, to plan future experimental studies on poorly described heteromeric nAChRs (see Table 3), and to give hints to evaluate the Ca^2+^ permeability of other receptor-channels, as for instance the highly relevant insect nAChRs, representing the major target for several insecticides (Dupuis et al., 2012) and never described in terms of *Pf*. Furthermore, in the future this approach could be useful to analyze other classes of ligand-gated channels, such as ionotropic glutamate receptors, whose Ca^2+^ permeability play a major role in synaptic plasticity and in neurodegenerative processes. The knowledge of the molecular mechanisms regulating Ca^2+^ entry through ion channels represents the first step to understand how to modulate it, in particular when and where the excessive accumulation of intracellular Ca^2+^ endangers cell health and survival.

## Author contributions

SF designed, wrote and approved this study, and agrees to be accountable for all aspects of the work in ensuring that questions related to the accuracy or integrity of any part of the work are appropriately investigated and resolved.

### Conflict of interest statement

The author declares that the research was conducted in the absence of any commercial or financial relationships that could be construed as a potential conflict of interest.
